# Microbial Contamination in Hospital Environment Has the Potential to Colonize Preterm Newborns’ Nasal Cavities

**DOI:** 10.3390/pathogens10050615

**Published:** 2021-05-17

**Authors:** Carolina Cason, Maria D’Accolti, Giuseppina Campisciano, Irene Soffritti, Giuliano Ponis, Sante Mazzacane, Adele Maggiore, Francesco Maria Risso, Manola Comar, Elisabetta Caselli

**Affiliations:** 1 Department of Advanced Translational Microbiology, Institute for Maternal and Child Health—IRCCS “Burlo Garofolo”, 34137 Trieste, Italy; carolina.cason@burlo.trieste.it (C.C.); giusi.campisciano@burlo.trieste.it (G.C.); giuliano.ponis@gmail.com (G.P.); 2 Department of Medical Sciences, University of Trieste, 34137 Trieste, Italy; 3 Department of Chemical and Pharmaceutical Sciences, Section of Microbiology and LTTA, University of Ferrara, 44121 Ferrara, Italy; maria.daccolti@unife.it (M.D.); irene.soffritti@unife.it (I.S.); elisabetta.caselli@unife.it (E.C.); 4 CIAS Research Centre, University of Ferrara, 44122 Ferrara, Italy; sante.mazzacane@unife.it; 5 Institute for Maternal and Child Health—IRCCS “Burlo Garofolo”, 34137 Trieste, Italy; adele.maggiore@asuits.sanita.fvg.it; 6 Department of Neonatology, Institute for Maternal and Child Health—IRCCS “Burlo Garofolo”, 34137 Trieste, Italy; francescomaria.risso@burlo.trieste.it

**Keywords:** healthcare associated infections, preterm newborns, antimicrobial resistance genes, microbiome, environmental microbial contamination

## Abstract

Infants born before 28 weeks are at risk of contracting healthcare-associated infections (HAIs), which could be caused by pathogens residing on contaminated hospital surfaces. In this longitudinal study, we characterized by NGS the bacterial composition of nasal swabs of preterm newborns, at the time of birth and after admission to the Neonatal Intensive Care Unit (NICU), comparing it with that of the environmental wards at the time of delivery and during the hospitalization. We characterized the resistome on the samples too. The results showed that environmental microorganisms responsible for HAIs, in particular *Staphylococcus* spp., *Streptococcus* spp., *Escherichia-Shigella* spp., and *K. pneumoniae*, were detected in higher percentages in the noses of the babies after 13 days of hospitalization, in terms of the number of colonized patients, microorganism amount, and relative abundance. The analysis of nasal bacteria resistome evidenced the absence of resistance genes at the time of birth, some of which appeared and increased after the admission in the NICU. These data suggest that hospital surface microbiota might be transported to respiratory mucosae or other profound tissues. Our study highlights the importance of a screening that allows characterizing the microbial profile of the environment to assess the risk of colonization of the newborn.

## 1. Introduction

Extremely preterm infants, born before 28 weeks, receiving care in a Neonatal Intensive Care Unit (NICU) are at high risk for contracting healthcare-associated infections (HAIs) [1,2]. HAIs are significant causes of morbidity and mortality in NICUs [3], with a prevalence that goes from 6% to 50% and mortality between 20% and 80%, depending on the risk factors [4,5]. 

The immaturity of newborns (so defined in the first 28 days of life) is linked to more immature immune responses, explaining their vulnerability to pathogens [6,7], which contributes to the occurrence of healthcare-associated infections. Invasive diagnostic and therapeutic procedures represent additional risk factors for pathogenetic NICU patients [6,8].

Neonatal infections are classified into early onset infections (occurring in the first 72 h of life) contracted at the time of delivery and late-onset infections, which occur after 72 h from birth, related to acquisition at home, or in hospital settings [9]. Among infants born preterm, early onset sepsis often represents a fatal illness, particularly among newborn infants of the lowest gestational age. Bacterial bloodstream infection is one of the most common events in hospitalized newborns [10], mainly caused by coagulase negative staphylococci [11].

Neonatal sepsis shares symptoms with other health conditions and is difficult to diagnose in the laboratory, which leads to a tendency to overdiagnose and treat this pathology. The extremely preterm infants with very low birth weight (VLBW) are treated with antibiotics, often for prolonged periods, in the absence of a confirmed infection [12,13,14,15]. Antibiotic exposures after birth are associated with multiple subsequent several negative outcomes, such as the development of asthma [16], allergies, prolonged hospitalization, multidrug-resistant bacterial infections [17], and even obesity later in life [18], making the risk/benefit balance of these approaches uncertain [16,17,18,19,20,21,22,23]. 

Nevertheless, the association between inadequate use of antibiotics and an increase of neonatal resistant bacterial HAIs in neonatal units has been reported [24]. 

For example, the main microorganisms responsible for hospital infections include Methicillin-resistant *Staphylococcus aureus* (MRSA), Carbapenem-resistant *Enterobacteriaceae*, Vancomycin-resistant *Enterococci* (VRE), and multidrug-resistant (MDR) *Clostridium difficile*, *Acinetobacter* spp., and *Pseudomonas aeruginosa* [25].

Additionally, fungi are involved, in particular invasive fungal infections due to *Candida* spp. are the third most common LOS in VLBW infants [26] with a higher incidence in NICU wards than in other paediatric or adult populations [27]. 

Among the main diseases associated with infections contracted in neonatal hospital settings, late onset sepsis (LOS) is considered the main cause of morbidity and mortality among preterm infants [28,29], with serious sequelae in surviving infants including necrotizing enterocolitis, bronchopulmonary dysplasia, and neurodevelopmental impairment [30]. A large proportion of all LOS is represented by central line-associated bloodstream infections (CLABSIs) [31]. LOS has been shown to be mainly caused by coagulase-negative staphylococci (CoNS) (39%), followed by *Escherichia coli* (9%) [32]. Other conditions frequently linked to HAIs in newborns are ventilator-associated pneumonia, mainly caused by Gram-negative bacteria [33] and ventricular shunt infections, mainly caused by Gram-positive organisms [34]. The risk of those pathologies increases with the low weight of the newborn, the gestational age, and the prolonging of the medical practice [26,35,36]. 

In recent years, there has been a growing interest in the role of the contaminated hospital environment in the transmission of HAIs [37]. Contaminated surfaces act as a reservoir for many pathogens, including those from the patients themselves [38], and can, therefore, be a substantial source for transmission of hospital infections [39,40]. Microorganisms can maintain their infectivity on dry inanimate surfaces even for weeks, and some spores can survive for months [41].

NICU surfaces harbour a large number of bacterial and fungal taxa, including members associated with HAIs in neonates. Recent studies have investigated the hypothesis that colonies of pathogenic agents on the surfaces of NICUs may increase the risk of infection for premature infants, without showing a species-specific causal association between environmental contamination and neonatal infection [42,43].

Recently, the approach based on next-generation sequencing (NGS) has emerged as a comprehensive and fast tool for disease surveillance and evolutionary analysis of infectious diseases [44,45]. By using NGS in this observational study we aimed to explore how the environmental microbiome can colonize the upper respiratory tract of preterm infants and which bacteria are most impactful in the clinical surveillance procedures usually adopted by the hospital. A multiple time-point analysis of nasal swabs of preterm newborns and environmental surface samples was performed simultaneously, collecting one set of samples at birth, and two set of samples during the period of hospitalization in the NICU.

## 2. Results

### 2.1. The Microbial Profile of Newborns Nasal Swabs

A longitudinal study was carried out analysing the bacterial composition of nasal swabs, of low-weight preterm newborns at birth (group N0) and after admission to the NICU (group N9 after 9 days, and group N13 after 13 days of permanence in the ward) comparing it with that of the environmental surfaces of the wards at the time of delivery and during the period of hospitalization.

### 2.2. NGS Analysis

**Figure 1:**
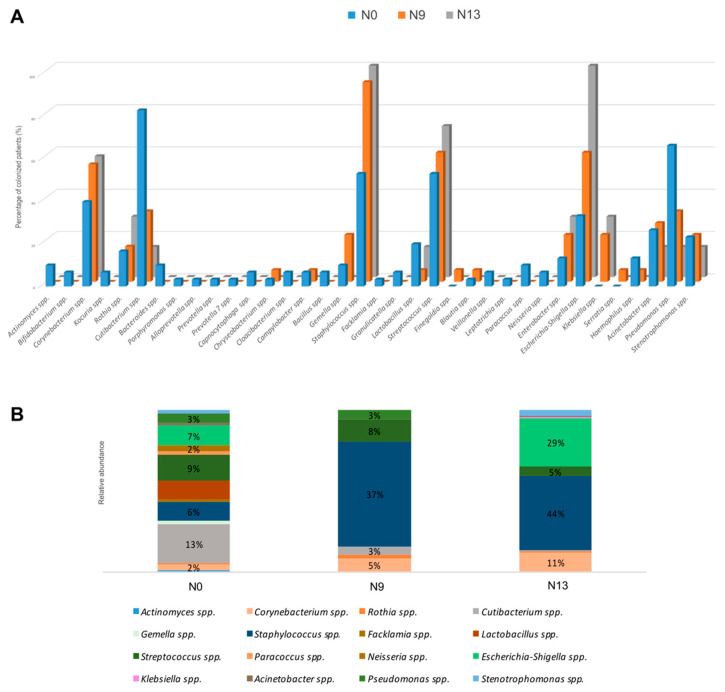
The most abundant bacterial communities of nasal swabs from newborns at time of birth and after two follow-ups. Results were obtained by NGS. (**A**) Percentage of colonized patients for each genus; (**B**) mean of bacterial relative abundances for each group. N0: samples collected at time of birth. N9: samples collected after 9 days of permanence in NICU. N13: sample collected after 13 days of permanence in NICU.

The analysis of nasal swabs by NGS led to the identification of bacterial genera *Corynebacterium* spp., *Staphylococcus* spp., *Streptococcus* spp., *Escherichia-Shigella* spp., *Acinetobacter* spp., *Pseudomonas* spp., *Klebsiella* spp., *Enterobacter* spp., *Lactobacillus* spp., *Cutibacterium* spp., *Stenotrophomonas* spp., *Haemophilus* spp., *Gemella* spp., and *Rothia* spp. as shown in Figure 1A. This figure reports the percentage of patients colonized by bacteria for each of the examined groups (N0, N9, and N13). *Corynebacterium* spp., *Staphylococcus* spp., *Streptococcus* spp., *Escherichia-Shigella* spp., *Klebsiella* spp., and *Enterobacter* spp. showed higher percentages of colonized patients in the N13 group, including newborns after 13 days of permanence in NICU. On the other hand, *Cutibacterium* spp. and *Pseudomonas* spp. had higher percentages in infants at the time of birth (N0). 

As for the average of the relative abundances for each genus within the three groups *Corynebacterium* spp., *Staphylococcus* spp., and *Escherichia-Shigella* spp. demonstrated an increase of relative abundance from the group N0 to the group N13: *Corynebacterium* spp. (N0: 2%; N9: 5%; N13: 11%), *Staphylococcus* spp. (N0: 6%; N9: 37%; N13: 44%), and *Escherichia-Shigella* spp. (N0: 7%; N9: 0%; N13: 29%) (Figure 1B). The observed differences were statistically assessed by Kruskal–Wallis test, showing significance concerning *Staphylococcus* spp. (FDR *p* = 0.047) and *Escherichia-Shigella* spp. (FDR *p* = 0.047). For these two bacterial genera there was an increasing trend both in the number of colonized subjects and in the relative abundance value (Figure 1).

### 2.3. Bacterial Composition of Vaginal Swabs in Pregnant Women

**Figure 2:**
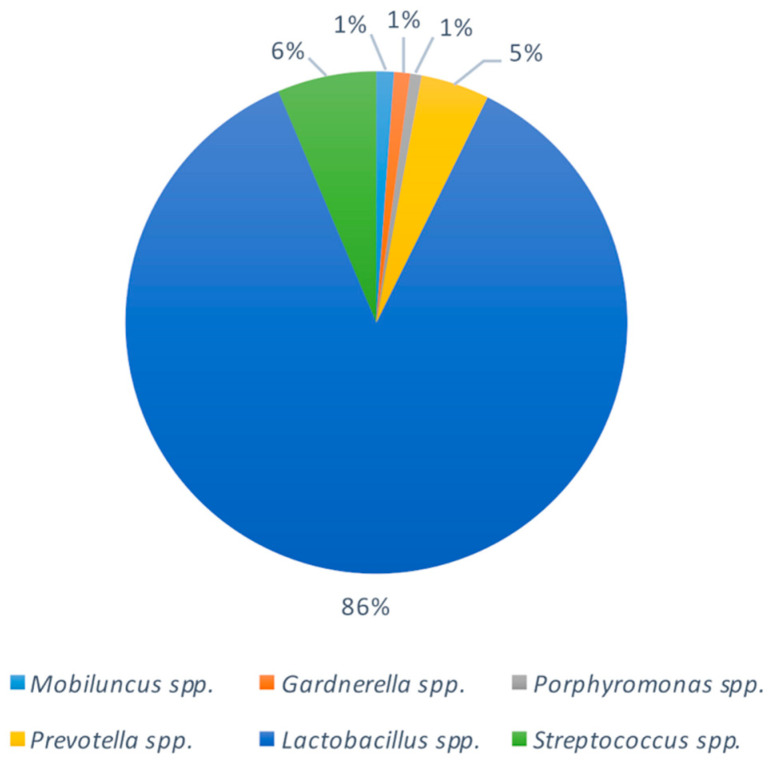
Predominant bacterial communities of vaginal swabs from pregnant women. Results were obtained by NGS. Data are represented as mean relative abundances.

To evaluate the contribution of vaginal bacteria in the colonization of the nasal microbiome of newborns, we analysed 20 vaginal swabs from women before giving birth in the delivery room (DR) (Figure 2). The detected bacteria were in line with previous data (Freitas et al., 2018; Fettweis et al., 2019). *Lactobacillus* spp., representing the predominant vaginal genus, was found with a relative abundance of 6% in 20% of newborns (N0), higher than the other groups considered. *Prevotella* spp. was detected only in 3% of N0 group, and *Streptococcus* spp. was detected with higher relative abundance in the N0 group (9% in the 53% of the babies of the group) than N9 and N13.

### 2.4. Impact of the Environmental Bacterial Microbiome on Nasal Colonization of Newborns

**Figure 3:**
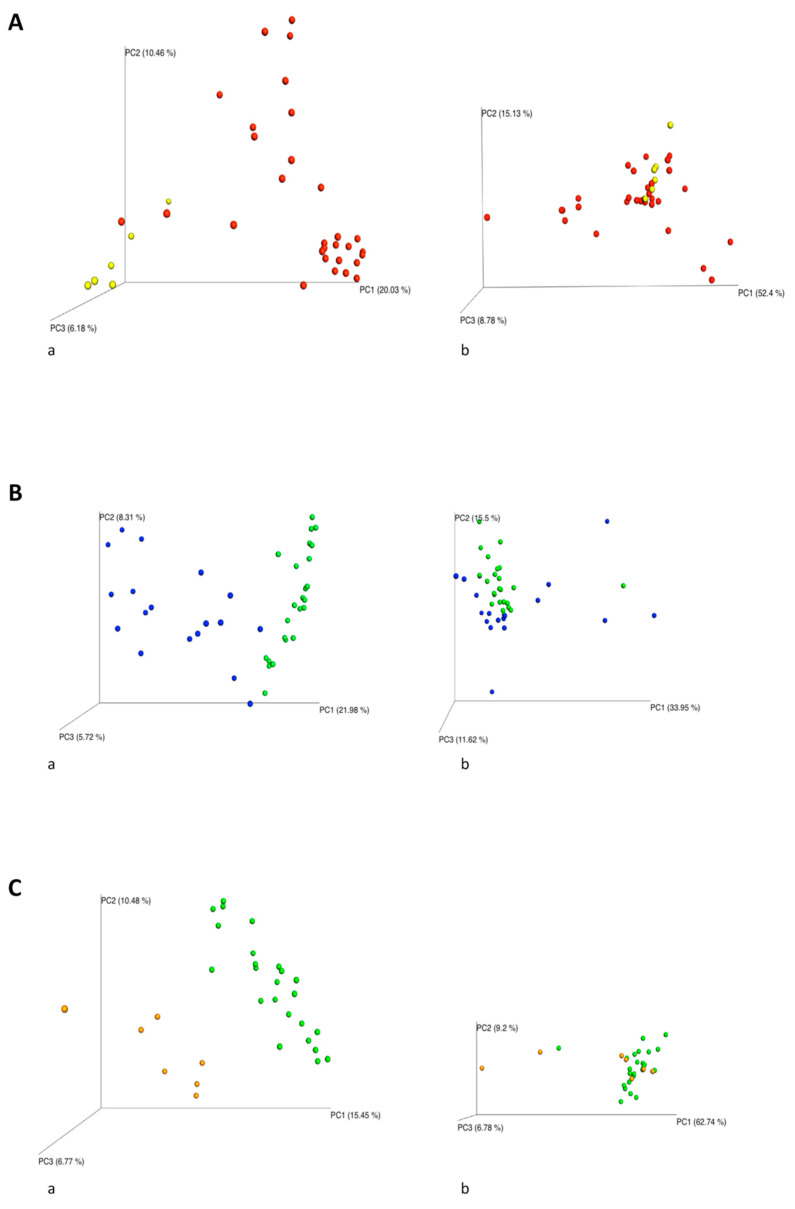
Emperor PCoA plot generated from the jackknifed_beta_diversity.py script of QIIME. Unweighted (a) and weighted (b) UniFrac-based PCoA, each dot represents a sample. (**A**) N0 vs. DR. N0 (red), DR (yellow). N0: nasal swabs collected at time of birth. DR: environmental samples from the Delivery Room. (**B**) N9 vs. NICU. N9 (blue), NICU (green). N9: nasal swabs collected after 9 days of permanence in NICU. NICU: environmental samples from the ward. (**C**) N13 vs. NICU. N13 (orange), NICU (green). N13: nasal swabs collected after 13 days of permanence in NICU.

We explored the influence of the environmental microbiome on newborns’ nasal colonization considering the environmental microbiome of the DR (N0 vs. DR) and of NICU, collected after baby’s permanence in this structure (N9 vs. NICU and N13 vs. NICU). The bacterial diversity was compared using the unweighted and weighted UniFrac distance matrices, the results are visualized by a Principal Coordinates Analysis (PCoA), both with weighted and unweighted UniFrac. A one-way Analysis of Similarity (ANOSIM) statistical test was applied to the UniFrac distance matrices to test the significant differences according to the clinical grouping. For the comparison between the group N0 and DR (Figure 3A), ANOSIM attributed a significant difference to the grouping for the unweighted UniFrac (*p* = 0.001, R = 0.9), but not for the weighted UniFrac (*p* = 0.8, R = −0.12). For the N9 and NICU groups (Figure 3B), ANOSIM attributed a significant difference to the grouping both for the unweighted (*p* = 0.001, R: 0.67) and weighted (*p* = 0.001, R: 0.27). For the groups N13 and NICU (Figure 3C), there was a significant difference both for the unweighted (*p* = 0.001, R: 0.82) and weighted (*p* = 0.008, R: 0.36) UniFrac. These tests show that the N0 and DR groups were very different from each other in terms of bacterial composition, while in terms of relative abundances there were some overlaps. The N9 vs. NICU and N13 vs. NICU groups showed more overlaps in terms of bacterial composition and relative abundances.

**Figure 4:**
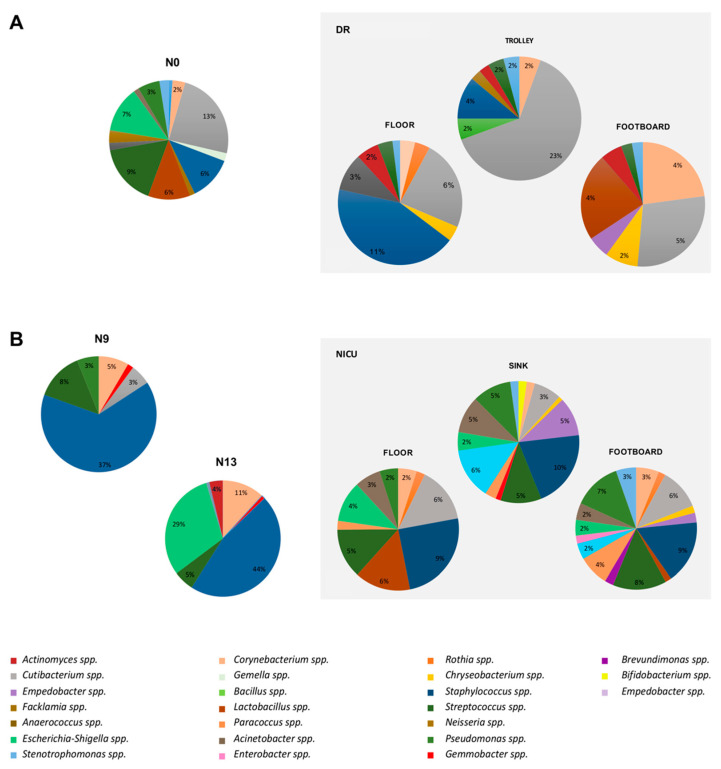
The predominant bacterial communities of nasal swabs from newborns in comparison with the environmental ones. Results were obtained by NGS. Data are expressed as mean relative abundance values. (**A**) N0 vs. DR. N0: nasal swabs collected at time of birth; DR: environmental samples of delivery room divided into type of surface (floor, footboard, and trolley). (**B**) N9 and N13 vs. NICU. N9: samples collected after 9 days of permanence in NICU. N13: sample collected after 13 days of permanence in NICU; NICU: environmental samples of NICU divided into type of surface (floor, footboard, and sink).

The bacterial composition of the selected surfaces was compared with that of the nasal swabs, to understand the significant source of infection, considering the mean of the relative abundances of each genus. Table 1 summarizes the bacteria found in the delivery and NICU rooms and colonizing the nasal microbiome of newborns. Notably, in the DR, *Cutibacterium* spp. represent the most frequent microorganisms found in the nose of newborns. On the other hand, in NICU, *Staphylococcus* spp. predominated both in N9 (94%, 12/13) and in N13 groups (100%, 7/7). Figure 4A shows the microbiome distribution of the DR in comparison with the nasal swabs of newborns of group N. *Cutibacterium* spp. was present at higher relative abundance (13%) in nasal swab compared to other genera, and it was detected at higher values in the medical trolley (23%) than the other points examined. *Staphylococcus* spp. showed a relative abundance of 6% in nasal swabs, this genus represented the primary contaminant of the floor of DR, with a relative abundance of 11%. *Lactobacillus* spp. and *Corynebacterium* spp., both with a relative abundance of 4% in the beds’ footboards, showed a rate of noise colonization of 6% and 2%, respectively.

We then compared the environmental microbiome of the NICU with the nasal microbiome of newborns admitted to the ward and in the two consecutive follow-up points (N9 and N13) (Figure 4B). *Staphylococcus* spp. was found to have the highest relative percentages both in nasal swabs of N9 babies (37%) and in the N13 group (44%). The major NICU source appeared to be the footboard and floor (9%), as well as the sink (10%). *Escherichia-Shigella* spp. was detected in 29% of the N13 group, and in the NICU was found mainly on the floor (4%). *Streptococcus* spp. had similar values in noses (N9: 8%, N13: 5%) and surfaces (floor and sink: 5%, footboard 8%). *Corynebacterium* spp., present in all three surfaces of the NICU in percentages less than 3%, had an average relative abundance of 5% in the N9 group and 11% in the N13 group. Other bacterial genera present in both nasal swabs and the NICU environment were: *Pseudomonas* spp. (S: 3%, footboard: 7%, floor: 2%, sink 5%) and *Stenotrophomonas* spp. (N13: 4%, footboard: 3%, sink: 1%).

### 2.5. Microarray Analysis Results

The results obtained by NGS were confirmed by the qPCR microarray analyses, which allowed the identification of bacteria up to species level, evidencing a progressive increase of positivity for specific microorganisms in the nasal swabs of newborns hosted in the NICU ward over time (N13 > N9 > N0). Within the genus *Staphylococcus*, the main species were *aureus* (N0: 3%, N9: 33%, N13: 43%) and *epidermidis* (N0: 27%, N9: 89%, N13: 100%). Instead, within the genus *Streptococcus*, the most frequently detected species were *pneumoniae*, *infantis*, *oralis*, and *salivarius*, with higher identification rates in the N13 group. This method also allowed the identification of the fungus *Candida albicans* in group N9 (6%) and N13 (43%), but not in group N0. Other microorganisms increasing in N9 and N13 compared to group N0 included *Enterococcus faecalis* (N0, 20%; N9, 55%; N13, 85%), *Escherichia-Shigella* (N0, 20%; N9, 38%; N13, 71%), *Klebsiella pneumoniae* (N0, 0%; N9, 44%; N13, 85%), *K. oxytoca/Enterobacter cloacae* (N0, 23%; N9, 67%; N13, 86%), *Pseudomonas aeruginosa* (N0, 7%; N9, 38%; N13, 43%), and, although to a less extent, *Acinetobacter baumannii* (N0, 13%; N9, 11%; N13, 28%).

Comparative quantitation between newborn groups evidenced significant increases over time (N13 vs. N0) of *S. epidermidis*, *K. pneumoniae/oxytoca*, *Escherichia-Shigella* (about 3 logs; *p* < 0.01), *E. faecalis*, *S. aureus*, *P. aeruginosa*, *S. pneumonia/infantis/oralis/salivarius*, and *C. albicans* (between 1 and 2 logs, *p* < 0.01). Interestingly, the results of microarray analysis performed on environmental samples of DR and NICU rooms showed that the highest contamination levels were ascribable to *S. epidermidis* (2200 genome copies per 100 cm^2^) and *K. pneumoniae/Enterobacter* (1833 genome copies per 100 cm^2^), followed by *P. aeruginosa* (186.7 copies/100 cm^2^), *S. aureus* (153.3 copies/100 cm^2^), *Enterococcus faecalis/faecium* (47.5 copies/100 cm^2^), and *A. baumannii* (20.3 copies/100 cm^2^). On the contrary, the other species were less represented. Contamination levels were higher on the floor and sink compared to bed footboard (not shown). In the DR, the level of contamination was generally lower compared to the NICU ward, including essentially *Staphylococci* (*S. epidermidis*, 310 copies/100 cm^2^; *S. aureus* 3.3 copies/100 cm^2^) and *E. faecium* (13.4 copies/100 cm^2^), mainly present on the floor and sink.

**Figure 5:**
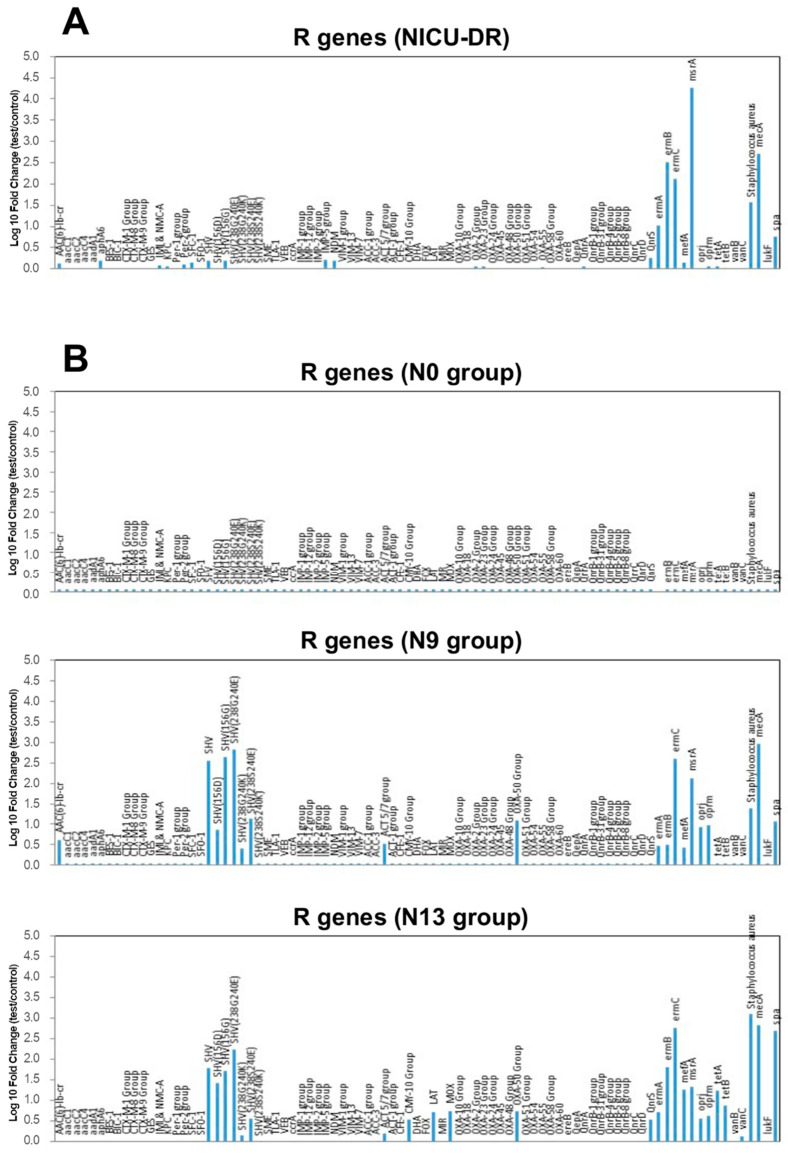
Resistome characterization of the microbial population contaminating NICU surfaces. Results were obtained by qPCR microarray as described in Methods and are expressed as log_10_ fold change of each detected R gene, compared with the negative controls (NTC). The plotted data represent the mean values of duplicate samples obtained in two environmental samplings for NICU environmental duplicate sampled points (**A**) and for nasal swabs of N0, N9, and N13 newborn groups (**B**).

To further characterize the environmental and nasal microbiome, we performed the analysis of the population resistome of both contaminating environmental microbiome and microbiome colonizing newborns’ nose. The analysis was carried out by a qPCR microarray, simultaneously detecting 84 different R genes. The results are summarized in Figure 5. Interestingly, the comparative analysis of the resistome of the newborns hosted in the NICU ward evidenced a progressive enrichment of strains harbouring R genes over time (Figure 5 and Table 2). While at birth, no R genes were detectable in the newborn nasal microbiome, after 9 (N9 group) and 13 days (N13 group) of hospitalization, the analysis revealed, respectively, the appearance and the increase of several R genes, most of which were also present in the NICU environment.

## 3. Discussion

This study investigated the potential influence of the environment microbiome on preterm newborns’ colonization admitted to the NICU. Nasal swabs of newborns were collected routinely as part of the HAIs prevention and control system of the hospital. Given the fragility of the patients, no further practices were introduced for the collection of swabs from other anatomical sites. The nasal cavities represent a highly accessible airway microbial community that recently was confirmed to have a pivotal role in human health and, to date, few studies focused on the microbiome of the nostrils of newborns [46,47]. The colonization of the upper respiratory tract is the gateway for lower tract infections. For example, in infants’ nasopharyngeal colonization with *Moraxella* spp. and *Haemophilus* spp. early in life is linked to the development of lower respiratory tract infections and consecutive atopic disease and future asthma [48]. Additionally, nasopharyngeal colonization with *S. pneumoniae* showed to be an important prerequisite to severe respiratory pneumococcal diseases such as sepsis, meningitis, and pneumonia [49]. Furthermore, the respiratory microbiota tract may have a role in the development respiratory tract and in shaping the local neonatal immune system [50].

The importance of the role of the mother’s microbiome in the colonization of the newborn is recognized, not only of the vaginal one, but also of the intestinal, cutaneous, and oral one [51,52,53,54]. Thus, besides the environmental contribution, in this study we also evaluated the contribution of mother’s vaginal bacteria in the colonization of the nasal microbiome of newborns. The surveillance analysis of the nose microbiome composition of newborns during the first hour after birth showed the presence of numerous bacterial taxa, likely derived from the mother’s resident vaginal microflora and bacteria colonizing the DR environment. The nasal cavities of newborns harboured bacterial species such as *Lactobacillus* spp. and *Streptococcus* spp. from the vaginal origin, and *Cutibacterium* spp., *Staphylococcus* spp. *Corynebacterium* spp., *Acinetobacter* spp., *Pseudomonas* spp., *Stenotrophomonas* spp., and *Rothia* spp. from DR environment surfaces, demonstrating how these bacteria begin part of the human microbiome during the first minutes after birth and showed a dynamic profile associated with the time of hospitalization.

In particular, our study showed that the frequency of colonization by specific opportunistic pathogenic genera responsible for HAIs [55,56,57], becomes predominant of nose microbiome with the time of permanence in intensive care. The quantitative molecular methods employed allowed the detection of microorganisms in higher percentages in the N13 group compared to the N0 group both in terms of the number of colonized patients, microorganism amount and relative abundance. This trend was observed in particular for *Staphylococcus*, both *aureus* and *epidermidis* species, *Streptococcus* spp., *Escherichia-Shigella* spp., *K. pneumoniae*, and *K. oxytoca/E. cloacae species*. These bacterial genera were among those most frequently detected on the NICU floor and bed platforms, at higher contamination levels.

To note, bacteria belonging to the genus *Staphylococcus* spp., detected in all N13 group patients, with the highest percentage of relative abundance when compared to the N9 and N13 groups, and the amount of the other bacteria identified, are responsible for the majority of late-onset infections in VLBW babies [56]. Methicillin-resistant *S. aureus* (MRSA), detected on the surfaces of the NICU and the nasal swabs of newborns after admission to the ward, is associated with significant mortality and morbidity, especially in very immature preterm neonates [58,59]. Approximately one out of five neonates colonized with MRSA may develop an infection, as prematurity has been identified to be the leading risk factor for MRSA colonization and subsequent infections [60]. MRSA colonized patients can shed into the environment their strain of *Staphylococcus* [61], which can survive in hospital dust for up to a year resisting desiccation [62]. Additionally, *S. epidermidis* has emerged as a predominant pathogen of neonatal late-onset sepsis in VLBW infants [63].

Similarly, *C. albicans*, which was not present in the nose of newborns at the time of birth, was instead detected after their admission in NICU. *Candida* infections contracted by nosocomial transmission [64,65,66] are one of the main cause of late-onset sepsis in VLBW preterm neonates [67].

Consistent with potent contamination of newborns by environmental and potentially pathogenic nosocomial microbes, the analysis of nasal bacteria resistome evidenced the absence of resistance (R) genes at detectability levels in the infants at the time of birth. On the contrary, we detected several R genes after admission to the NICU, and they increased with the prolonged hospitalization. Most of these genes were also detected in the microbial population contaminating the ward environment, including beta-lactam resistance genes (SHV), quinolone resistance genes (QnrS), and macrolide resistance genes (ermA, ermB, ermC, mefA, msrA). Based on kinetic of newborns colonization by environmental microorganisms [68,69], based on the so-called paradigm of the “sterile womb”, with a few taxa present at birth, the absence of R genes at time 0 and their appearance at 9 and 13 days after birth are compatible with the arrival of resistant strains from the environment and their proliferation/colonization in the nasal cavities of the babies.

This data provides further support of environmental microorganisms colonization of the newborn patients, highlighting, once again, the role of the hospital environment as a source of potentially pathogenic ad drug-resistant microorganisms, with consequent difficulties in their containment and the management of the correlated infections [70,71].

The results of this study highlight the importance of active monitoring of the environmental microbial contamination, using high sensitivity molecular methods that guarantee the characterization of microbial communities in the wards as well as in admitting patients, allowing to understand the transmission routes through which environmental microbes can come in contact with patients. The ward contamination can contribute to the transmission of HAIs, and that environmental monitoring and microbiological surveillance can reduce the rate of infections [72]. The knowledge of the main microbial species, as well as the intrinsic genes of antibiotic resistance patterns, can guide more effective therapeutic treatment [73,74].

Furthermore, data from the screening of surfaces should be evaluated to generate increased or targeted cleaning approaches to prevent possible outbreaks [75] suggesting the implementation of new strategies for surface sanitizing. Most of the detergents used in sanitizing hospitals, based on chemical germicides, are not able to stably decontaminate the surfaces, as they cannot counteract the recontamination phenomena, ultimately responsible for the persistence of microorganisms in the environment [76,77]. Furthermore, chemical-based disinfectants, such as those based on chlorine also used for the sanitization of the surfaces of the hospital involved in this study, have a high environmental impact, and some of them have been reported to favour the selection of resistant strains, an undesirable side effect [78,79,80]. Among the proposed innovative methods, a sanitation system allowing colonization by benign microbes rather than potential pathogens appears potentially attractive, as successfully reported in a recent Italian multicentre study [81,82].

In conclusion, despite the pilot features of our study, the results show that environmental hospital microbes (including drug-resistant strains) detected on ward surfaces can reach the respiratory tract of preterm newborns starting from birth and increasing in the NICU. The data from this observational study suggest that hospital surface microbiota could be transmitted by contact, and it might also be transported, perhaps by air particles, even to respiratory mucosae or other profound tissues.

Based on these observations, we think that monitoring the hospital environment should be a mandatory aspect of infection prevention, control strategies, and suggests that molecular methodologies are a suitable tool to reach the goal of controlling potentially pathogenic contamination.

## 4. Materials and Methods

### 4.1. Patients and Samples

As part of the hospital infection control protocol, from November 2018 to January 2019, a total of 55 nasal swabs were collected from preterm infants, attending the NICU at the IRCCS Burlo Garofolo Hospital in Trieste, Italy, regardless of their clinical condition. The study time-course of sample collection included: 30 swabs at the time of birth (group N0), 18 after 9 days (group N9), and 7 after 13 days of permanence in the NICU ward (group N13) depending on the health status of the newborns. N9 and N13 groups include the newborns that from N0 group were hospitalized for 9 and 13 days, respectively. At the same time, four randomized rooms in the NICU and one in the DR were selected for environmental surveillance following a standard protocol [81]. A total of 24 samples from critical points, including the floor, footboard, and sink, were gathered from the NICU, while 6 were collected from floor, footboard, and hospital trolley of the DR. Sampling was performed twice and analysed by NGS and by a real time quantitative PCR microarray approach. Twenty vaginal swabs were additionally collected from pregnant women before giving birth in the DR.

### 4.2. Nasal Swab Collection

Anterior nasal swabs were collected by nurses and put in a sterile medium, using eSwabs and liquid amies transportation medium (Copan, Brescia, Italy). Immediately after collection, swabs were sent to the laboratory and stored at −80 °C until analysis. 

### 4.3. Environmental Sampling

Sterile rayon swabs (Copan, Brescia, Italy) were premoistened in a sterile saline solution and used to collect a surface of 100 cm^2^ delimited by a sterile 10 × 10 cm disposable plastic template (Copan, Brescia, Italy). Swabs were then put in 300 µL of sterile saline solution and stored at −80 °C until analysis. The samples were collected in duplicate and extracted separately.

### 4.4. Vaginal Swabs Collection

Samples were collected using a 200 mm polyethylene Cervix brush device (Rovers Medical Devices B.V., The Netherlands) under speculum examination, by a 360° rotation of the brush. Samples were then suspended in 1.5 mL of TE buffer and stored at −80 °C until analysis. 

### 4.5. Next Generation Sequencing Analysis

#### 4.5.1. DNA Extraction 

Before being processed, all samples were defrosted and vortexed. The DNA extraction was performed from 300 µL of the samples at a final elution volume of 100 µL for nasal and vaginal swabs, and in 50 µL for the environmental samples, using the automatic extractor Maxwell CSC DNA Blood Kit (Promega, Madison, WI, USA), according to the manufacturer’s instruction.

#### 4.5.2. Library Preparation 

The 16S rRNA gene (V3 region) was sequenced to characterize the composition of bacterial communities of samples. Alongside, negative controls, including no template controls (NTC), were processed. In addition, the swabs and reagents used for sampling were tested for the presence of bacterial DNA, confirming them to be sterile. A real time quantitative EvaGreen^®^ dye (Fisher Molecular Biology, Waltham, MA, USA) PCR was performed to amplify several bacterial species, using the U534R primer and the degenerated primer 27FYM (5′-AGR GTT YGA TYM TGG CTC AG-3′), targeting the V1-V3 region, (500 bp). A nested PCR was subsequently carried out with the primers B338F_P1-adaptor (B338F 5′-ACTCCTACGGGAGGCAGC-3′) and U534R_A_barcode (U534R 5′-ATTACCGCGGCTGCTGG-3′), targeting the V3 region (200 bp) of the 16S rRNA gene, with a different barcode for each sample attached to the reverse primer [83].

The PCR reactions were performed using the Kapa HiFi Hotstart ready mix 2X (Kapa Biosystems, Massachusetts, MA, USA) and BSA 400 ng/μL. We did this according to these conditions: initial denaturation step of 95 °C for 5 min, followed by a denaturation step at 95 °C for 30 s, annealing at 59 °C (V1-V3 PCR)/57 °C (V3 PCR) for 30 s, and extension at 72 °C for 45 s with maximum of 27 cycles for the V1-V3 PCR and a maximum of 13 cycles for the V3 PCR. Final extension step of 10 min at 72 °C. The correct size of the amplicon (260 bp) was assessed on a 2% agarose gel. 

The quantification of the amount of DNA was performed using a Qubit^®^ 2.0 Fluorimeter (Invitrogen, Carlsbad, CA, USA), and 100 ng of PCR from each sample was mixed to generate the pooled library and diluted to a concentration of 100 pM, according to manufacturer’s instruction. Ion PGM Hi-Q View OT2 200 kit was used to prepare the template on the Ion OneTouch™ 2 System (Life Technologies, Gran Island, New York, NY, USA). Sequencing was performed with the Ion PGM™ System technology using the Ion PGM Hi-Q View sequencing kit (Life Technologies, New York, NY, USA).

#### 4.5.3. Data Processing

The sequence data were processed using Quantitative Insights Into Microbial Ecology (QIIME 2 2020.2) [84]. High quality sequences (Q > 25) were demultiplexed and filtered with default parameters, except for the length (150 bp). Any sequence with ambiguous bases or a homopolymer length > 8 was removed. Further analysis was performed on a random subset of 2000 reads/sample. Taxonomy assignment at the genus level was performed against the reference taxonomy database SILVA V.132 [85] with a similarity threshold of 97%.

### 4.6. qPCR Analyses

#### 4.6.1. DNA Extraction 

Frozen environmental samples were thawed and vortexed to detach cells from swabs, Total DNA was extracted by a commercial kit (Gene All, Tema Ricerca, Italy), following the manufacturer’s instruction adjusted to optimize the extraction from Gram-positive bacteria, as already described [86].

#### 4.6.2. Microarray Analysis

Characterization of the microbial contamination at the species level was performed by a customized array assessing simultaneously the presence of 22 bacterial and mycetes species, among the most frequently involved in HAIs as previously described [45,86]. *Acinetobacter baumanii*, *Aspergillus fumigatus*, *Candida albicans*, *Citrobacter freundii*, *Clostridium difficile*, *Clostridium perfrigens*, *Enterobacter cloacae/Klebsiella oxytoca*, *Enterococcus faecalis*, *Enterococcus faecium*, *Escherichia coli*, *Klebsiella pneumoniae*, *Proteus vulgaris* and *mirabilis*, *Pseudomonas aeruginosa*, *Staphylococcus aureus* and *epidermidis*, *Streptococcus agalactiae*, *anginosus*, *prneumoniae*, *pyogenes*, *infantis/oralis*, and *salivarius* (Qiagen, Hilden, Germany). A pan-bacterial (panB) and pan-mycetes (panM) quantification, as well as a positive amplification control (PPC) and negative no template control (NTC) were also included in the array. The use of a real-time microarray, together with the normalization based on panB/panM results, allowed relative quantification of each parameter.

The analysis of microbial contamination at the environmental level was performed by a similar customized qPCR microarray, including all the previously mentioned species except for Streptococci (Qiagen, Hilden, Germany).

Characterization of antimicrobial resistance (AMR) genes of the contaminant population was obtained by a microarray detecting and quantifying simultaneously 84 different AMR genes (Qiagen, Hilden, Germany), as previously described [45,81,82,87]. 

### 4.7. Statistical Analyses 

Statistical analyses were performed with QIIME 2 (2020.2). Beta diversity (between sample-diversity comparison) was assessed with weighted and unweighted UniFrac distance matrices [88] and presented with principal coordinates analysis (PCoA). Analyses of Similarities (ANOSIM) and Kruskal–Wallis tests were performed to compare the community composition in the group considered (N0-N9-N13, N0-AP, N9-AT, N13-AT), assuming a statistically significant FDR *p* value of at least < 0.05.

For microarray results, statistical analyses were performed using parametric (Student’s *t* test) and non-parametric (Mann–Whitney) tests, assuming a statistically significant *p* value of at least <0.05. Bonferroni correction for multiple comparisons was applied for the analysis of microarray data (a *pc* value < 0.05 was considered significant). 

## Figures and Tables

**Table 1:** Main bacterial genera of environment compared with nasal swabs. N0: nasal swabs collected at time of birth; N9: samples collected after 9 days of permanence in NICU. N13: sample collected after 13 days of permanence in NICU. Data are expressed as percentage of colonized patients for each genus.

Main Bacterial Genera of DR	N0 Colonized Patients (%)	Main Bacterial Genera of NICU	N9 Colonized Patients (%)	N13 Colonized Patients (%)
*Staphylococcus* spp.	53%	*Staphylococcus* spp.	94%	100%
*Streptococcus* spp.	53%	*Streptococcus* spp.	61%	71%
*Cutibacterium* spp.	83%	*Cutibacterium* spp.	33%	14%
*Corynebacterium* spp.	40%	*Corynebacterium* spp.	56%	57%
*Escherichia-Shigella* spp.	33%	*Escherichia-Shigella* spp.	61%	100%
*Acinetobacter* spp.	67%	*Acinetobacter* spp.	33%	14%
*Pseudomonas* spp.	67%	*Pseudomonas* spp.	33%	14%
*Stenotrophomonas* spp.	23%	*Stenotrophomonas* spp.	22%	14%
*Rothia* spp.	17%	*Rothia* spp.	17%	19%
*Lactobacillus* spp.	20%	*Lactobacillus* spp.	6%	14%
*Chryseobacterium* spp.	3%	*Chryseobacterium* spp.	6%	/
*Paracoccus* spp.	10%	*Paracoccus* spp.	/	/
*Actinomyces* spp.	10%	*Enterobacter* spp.	22%	29%
*Gemella* spp.	10%	*Bifidobacterium* spp.	/	/
*Bacillus* spp.	7%	*Alishewanella* spp.	/	/
*Neisseria* spp.	7%	*Brevundimonas* spp.	/	/
*Facklamia* spp.	3%	*Gemmobacter* spp.	/	/
*Empedobacter* spp.	/	*Empedobacter* spp.	/	/
*Anaerococcus* spp.	/			

/: No detection.

**Table 2:** Presence of AMR-associated genes in the nasal microbiota of newborns in NICU ward.

R Genes	N0 Group	N9 Group	N13 Group
AAC(6)-lb-cr	−	+	−
SHV group	−	+	+
ACT 5/7 group	−	+	+
CMY-10 group	−	−	+
LAT	−	−	+
MOX	−	−	+
OXA-50 group	−	+	+
QnrS	−	−	+
ermB	−	−	+
ermC	−	+	+
mefA	−	−	+
msrA	−	+	+
oprj	−	+	+
oprm	−	+	+
tetA	−	−	+
vanC	−	−	+
mecA/spa	−	+	+

−: No detection of the resistance gene; +: Presence of the resistance gene.

## Data Availability

The data presented in this study are available on request from the corresponding author.
